# Application of Graphitized Multi-Walled Carbon Nanotubes Combined with Orbitrap High-Resolution Mass Spectrometry for the Rapid Detection of Ten Toxins in Wild Mushrooms

**DOI:** 10.3390/toxins17090445

**Published:** 2025-09-04

**Authors:** Bo Zhang, Yang Liu, Shengnan Li, Ruonan Li, Yunhui Zhang, Hua Zhao

**Affiliations:** 1 College of Basic Medicine, Zunyi Medical University, Zunyi 563000, China; bozhangzmu@163.com; 2 School of Public Health, Zunyi Medical University, Zunyi 563000, China; 3 Zunyi Center for Disease Control and Prevention, Zunyi 563000, China

**Keywords:** wild mushrooms, toxins, graphitized multi-walled carbon nanotubes

## Abstract

Wild mushroom poisoning is an emerging global food safety issue, especially in subtropical regions like southwestern China, where incidents are geographically clustered. Current detection methods are often time-consuming and overlook region-specific toxins. We developed a rapid, sensitive, and accurate method for the simultaneous detection of ten characteristic mushroom toxins prevalent in Guizhou, China. The method combines graphite multi-walled carbon nanotubes (G-MWCNTs) for sample preparation with Orbitrap high-resolution mass spectrometry (HRMS). Wild mushroom samples were extracted via ultrasonic-assisted methanol–water extraction, purified using G-MWCNTs, and separated on a Hypersil GOLD C18 column (100 mm × 2.1 mm, 1.9 μm). Gradient elution was performed with 0.1% formic acid + 0.01% ammonia and acetonitrile; quantification used the external standard method. The method achieved LODs of 0.005–0.2 mg/kg and LOQs of 0.015–0.6 mg/kg, with RSDs below 18.11% and excellent linearity (R^2^ = 0.9936–0.9989). Among 45 wild mushroom samples, toxin levels ranged from 0.032 to 445.10 mg/kg, with a detection rate of 22.22%, suggesting notable poisoning risk. This method reduces pretreatment time while ensuring high analytical performance, offering a reliable tool for rapid toxin screening and supporting regional surveillance of wild mushroom poisoning.

## 1. Introduction

Wild mushrooms represent a vital natural food source with distinctive sensory qualities, high nutritional density, and potential pharmacological activities, attracting increasing interest in both nutrition and biomedical research [1,2]. While widely consumed for their health benefits and functional properties, especially through wild harvesting [3,4], certain wild species pose severe health hazards due to their content of toxic compounds such as amatoxins, muscarine, and psilocybin. These toxins can induce hepatotoxicity, nephrotoxicity, gastrointestinal inflammation, neuropsychiatric disorders, and can be fatal in some cases [5]. More than 16,000 mushroom species have been identified globally, with over 4000 known in China, including over 400 toxic varieties [6]. In many parts of China—particularly in mountainous regions like Sichuan, Yunnan, and Guizhou—wild mushroom consumption is culturally entrenched. However, the morphological similarities between toxic and edible species frequently result in misidentification, as traditional methods based on local knowledge lack accuracy. Consequently, poisoning incidents occur annually at a high rate. Epidemiological data indicate that mushroom poisoning is now one of the leading causes of foodborne disease outbreaks and mortality in China [7,8].

Mushroom toxins encompass a diverse array of chemical entities and are commonly classified based on their toxic constituents, including muscarine, amatoxins, ibotenic acid gyromitrin, orellanine, psilocybin, gastrointestinal irritants, isoxazoles, and others [9,10]. Timely and accurate diagnosis of mushroom poisoning is pivotal in clinical practice, particularly in acute intoxication scenarios where rapid intervention can significantly reduce patient morbidity and mortality. The clinical severity of poisoning is strongly associated with the concentration and type of toxin ingested. Therefore, precise identification of the mushroom species and its toxicological profile is critical for clinical decision-making. As the primary toxic agents, mushroom toxins must be characterized early to guide physicians in developing targeted therapeutic strategies [8,11,12,13]. At present, the identification of poisonous mushrooms relies predominantly on expert mycological analysis, involving morphological and microscopic characterization. However, such expertise is typically absent in most primary healthcare settings [8,14]. Complicating matters further, residual mushroom samples retained by patients are often subjected to cooking or processing, which hampers morphological identification. Nevertheless, most toxins are heat-stable and retain their bioactivity post-cooking. As such, the establishment of a rapid, reliable, and accurate method for toxin detection is of paramount importance for guiding clinical treatment, executing timely emergency interventions, and improving patient survival outcomes.

Mushroom toxin detection primarily employs analytical techniques such as enzyme-linked immunosorbent assay (ELISA), capillary electrophoresis, high-performance liquid chromatography (HPLC), and liquid chromatography–tandem mass spectrometry (LC-MS/MS) [15]. ELISA allows for rapid screening; however, it is constrained by its limited toxin detection spectrum (typically 3–5 toxins per assay) and is insufficient for capturing the chemical diversity of mushroom toxins. Additionally, the development of specific antibodies for mushroom toxins is both time-intensive (6–12 months) and technically demanding [16]. Capillary electrophoresis is limited by low reproducibility, inadequate sample throughput, and suboptimal sensitivity, with detection limits up to 1 µg/mL [17]. HPLC, necessitates labor-intensive pretreatment procedures involving extraction and purification, which increases the risk of toxin degradation or loss. Moreover, it demonstrates limited specificity, poor stability over time, and is susceptible to matrix effects, all of which can adversely impact the accuracy and reliability of analytical results [18].

Liquid chromatography–tandem mass spectrometry (LC-MS/MS) has become a widely adopted method for the detection of mushroom toxins, attributed to its high analytical sensitivity and specificity [19]. More recently, ultra-high-performance liquid chromatography coupled with quadrupole-Orbitrap high-resolution mass spectrometry (UPLC-Q Orbitrap HRMS) has demonstrated enhanced capabilities by integrating the high selectivity of quadrupole precursor ion selection with the precise mass accuracy of Orbitrap analyzers. This allows for accurate molecular and ionic characterization of mushroom toxins. Moreover, the acquisition of high-resolution MS/MS spectra facilitates the identification of unknown compounds with greater precision and confidence compared to traditional LC-MS/MS methods. Orbitrap-based HRMS has shown exceptional utility in the detection and identification of toxicants in emergency response scenarios, where rapid and accurate analysis is essential [20].

Despite the high sensitivity and selectivity of high-resolution mass spectrometry (HRMS), matrix interference remains a major obstacle in the accurate detection of target compounds using chromatographic techniques. Effective sample pretreatment is thus essential to reduce matrix effects, avoid the introduction of impure extracts, and improve the detection limits of analytes [21,22].

Currently, solid-phase extraction (SPE) and dispersive solid-phase extraction (dSPE) are the two major techniques employed for sample pretreatment and purification in mushroom toxin analysis. SPE, recognized as a pivotal method for biological sample purification, has gained widespread use for isolating and enriching trace toxins from complex matrices owing to its remarkable selectivity and purification capacity. Depending on different retention mechanisms, a variety of SPE sorbents have been successfully applied in amatoxin determination [23,24]. Nevertheless, traditional SPE is still constrained by its cumbersome workflow—which involves multiple steps such as activation, sample loading, washing, and elution—as well as its time-consuming nature [23]. By contrast, dSPE offers a much simplified process by dispersing sorbents directly into the sample solution, reducing the total handling time to less than 30 min. However, its purification efficiency remains unstable [25]. As such, the choice of sorbent is crucial for optimizing dSPE performance. Current strategies mainly follow two directions: (i) in-house synthesis of functionalized sorbents [26], which allows for selective adsorption of target analytes through surface functional group modification, but is limited by complex synthesis steps, lengthy preparation cycles, and the high cost of raw materials and purification reagents; (ii) the use of graphitized multi-walled carbon nanotubes (G-MWCNTs), which, compared to conventional C18 or PSA sorbents, exhibit unique physicochemical properties including π-π conjugated interactions, highly ordered carbon layers formed by graphitization, and a large specific surface area. These features enable stronger interactions with target compounds and more efficient removal of complex matrix interferences [27].

Carbon nanomaterials have recently gained attention for use in sample preparation due to their remarkable adsorption properties and chemical robustness. Multi-walled carbon nanotubes (MWCNTs), composed of concentric graphene cylinders, possess large surface areas, high aspect ratios, and modifiable surface sites, making them suitable for selectively binding matrix components and purifying analytes [27,28,29]. Among them, graphitized MWCNTs (G-MWCNTs), functionalized derivatives of MWCNTs, exhibit superior adsorption capacity and enhanced chemical stability due to the introduction of specific functional groups. These attributes make G-MWCNTs highly effective in extracting and concentrating target analytes from complex matrices [30,31]. To date, MWCNTs have been widely applied in combination with LC-MS/MS for residue detection in fields such as pesticides and veterinary drugs [32]. However, no existing studies have reported the integration of G-MWCNTs with Orbitrap HRMS for mushroom toxin detection. Developing such a method would not only represent a valuable advancement in analytical toxicology but also offer novel technical solutions for ensuring food safety in the context of wild mushroom consumption.

Accordingly, this study focused on detecting 10 key mushroom toxins frequently implicated in poisoning events in southwestern China, particularly Guizhou Province. The selected toxins include six lethal amatoxins (α-amanitin, β-amanitin, γ-amanitin, phalloidin, phalloidin, Phallisacin), two neurotoxins with distinct clinical profiles (muscarine and muscimol), the hemolytic compound gyromitrin, and the nephrotoxic compound orellanine. A novel rapid analytical approach was established by integrating graphitized multi-walled carbon nanotubes (G-MWCNTs) with Orbitrap high-resolution mass spectrometry, enabling the simultaneous detection of these toxins in wild mushroom samples. Through systematic optimization of sample pretreatment protocols and mass spectrometric parameters, the method demonstrated efficient toxin extraction, rapid chromatographic separation, and precise quantification.

## 2. Results

### 2.1. Selection of Liquid Chromatography Conditions

In liquid chromatography, the mobile phase typically consists of an organic phase and an aqueous phase. Acetonitrile and methanol are commonly used as organic solvents. In this study, a 2 mg/L mixed standard solution was used to compare the separation efficiency and chromatographic peak shapes of 10 mushroom toxins using either acetonitrile or methanol as the organic phase. The results showed that when methanol was used, α-amanitin, β-amanitin, and γ-amanitin exhibited poor peak shapes and peak splitting, while orellanine showed pronounced peak tailing. These effects may be attributed to the lower elution strength of methanol compared to acetonitrile, leading to peak distortion. In contrast, using acetonitrile as the organic phase produced well-shaped chromatographic peaks for all 10 mushroom toxins, with no splitting or tailing observed.

Further comparisons were conducted using three aqueous mobile phase compositions: 0.1% formic acid aqueous solution, 0.01% ammonium hydroxide aqueous solution, and a combination of 0.1% formic acid + 0.01% ammonium hydroxide aqueous solution. The combination of 0.1% formic acid and 0.01% ammonium hydroxide yielded the highest ion response, best separation, and optimal sensitivity for all 10 mushroom toxins. In contrast, when either 0.1% formic acid or 0.01% ammonium hydroxide was used alone, lower signal intensities and poorer peak shapes were observed.

**Figure 1:**
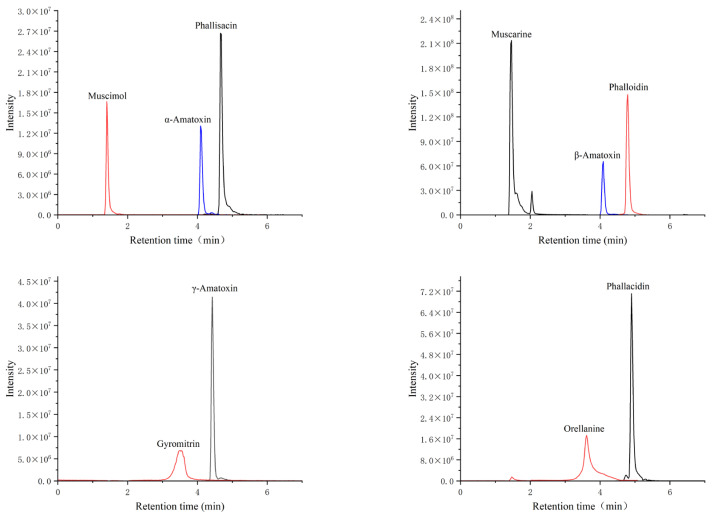
Extracted ion chromatograms of 10 mushroom toxins.

Based on these findings, acetonitrile was selected as the organic phase and the mixture of 0.1% formic acid + 0.01% ammonium hydroxide aqueous solution as the aqueous phase. The extracted ion chromatograms of the 10 mushroom toxins are shown in Figure 1, and all compounds were successfully separated within 9 min. The corresponding retention times are listed in Table 1.

### 2.2. Optimization of Mass Spectrometry Conditions

The high-resolution quadrupole-Orbitrap mass spectrometer enables accurate mass determination of target compounds. Data acquisition was performed in Full MS/dd-MS^2^ mode, in which full-scan MS data are first acquired, followed by automatic data-dependent MS^2^ fragmentation (CID, collision-induced dissociation) of identified precursor ions. To ensure the reliability of detection, MS^2^ acquisition was triggered only when the precursor ion signal intensity exceeded 1 × 10^3^. To further improve mass accuracy, the isolation window for precursor ions was set to 1 *m*/*z*.

Amanitins are cyclic octapeptides containing polar functional groups such as hydroxyl and amino groups, and can be ionized in both positive and negative ionization modes. Experimental results showed that α-amanitin, β-amanitin, and γ-amanitin exhibited higher protonation efficiency and stronger ion responses in the negative ion mode ([M − H]^−^), while the remaining seven mushroom toxins exhibited higher ion intensities in the positive ion mode ([M + H]^+^). Therefore, UPLC-Q-Orbitrap HRMS was operated in polarity-switching mode to simultaneously acquire both positive and negative full-scan spectra. Extracted ion chromatograms (EICs) were generated using the theoretical exact mass values of the compounds to obtain [M + H]^+^ or [M − H]^−^ signals.

The relative mass deviations of the exact masses for all 10 mushroom toxins were less than 0.75 × 10^−6^, indicating excellent mass accuracy. The specific mass deviation values and the most abundant product ions (dd-MS^2^) for each compound are listed in Table 1.

### 2.3. Optimization of Purification Sorbent Amount

This study investigated the effect of different amounts of graphitized multi-walled carbon nanotubes (G-MWCNTs) (50, 100, 150, 200, 250, 300, 350, 400, 450, and 500 mg) on the spiked recovery of 10 mushroom toxins. A blank matrix was spiked with 1 mg/kg of mixed mushroom toxin standards and processed according to the pretreatment method described in Section 5.4. Each concentration level was analyzed in triplicate.

The results showed that when 150 mg of G-MWCNTs was used, the recoveries of the 10 mushroom toxins ranged from 83.7% to 108.3%. However, increasing the sorbent amount to 200 mg resulted in a wider variation in recoveries (43.1% to 117.3%), and a general downward trend in recovery was observed with further increases in the amount of G-MWCNTs. This decline was particularly significant for phallacidin, phallisacin, and phalloidin—three highly polar compounds with strong hydrogen-bonding capabilities—which were more readily adsorbed by the G-MWCNTs. When the sorbent amount reached 400 mg, these three toxins were almost completely adsorbed and could not be detected.

**Figure 2:**
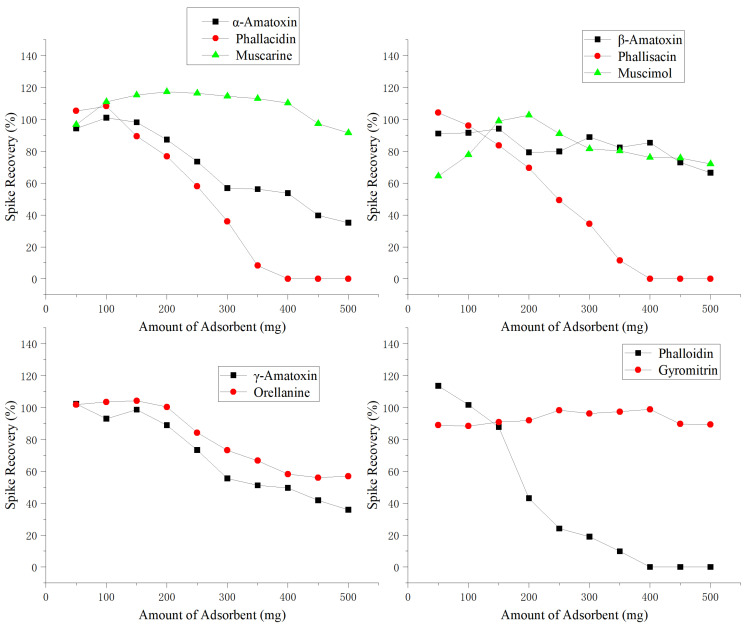
Effect of graphitized multi-walled carbon nanotube dosage on the recovery rates of 10 mushroom toxins.

Considering the overall recovery performance for all 10 toxins, 150 mg was determined to be the optimal amount of G-MWCNTs. As shown in Figure 2, spiked recoveries exceeded 83% for all toxins at this sorbent level. According to relevant standards, acceptable recovery rates for analytes spiked in the range of 0.1–10 mg/kg should fall within 80–110% [33]. Therefore, 150 mg of G-MWCNTs was selected as the optimized purification material to ensure the accuracy of the method.

### 2.4. Linearity, Limits of Detection and Quantification

Based on the spiking recovery experiments and following the sample pretreatment and detection procedures described in Section 5.5, the method’s limit of detection (LOD) and limit of quantification (LOQ) were calculated through statistical analysis of the experimental data. Standard calibration curves were constructed by plotting the concentrations of the prepared standards on the x-axis and the extracted ion chromatographic peak areas of the target compounds on the y-axis, from which linear regression equations and correlation coefficients were obtained. Detailed results are shown in Table 2. The results demonstrated that all 10 mushroom toxins exhibited good linearity within their respective concentration ranges, with correlation coefficients (R^2^) greater than 0.993. The LODs ranged from 0.005 to 0.2 mg/kg, and the LOQs ranged from 0.015 to 0.6 mg/kg.

### 2.5. Evaluation of Matrix Effect

During UPLC-Q Orbitrap HRMS analysis, matrix effects (ME) are commonly encountered. ME can lead to either signal suppression or enhancement of target compounds. When both the analyte and sample matrix components enter the LC-MS system simultaneously, matrix interferences may affect the ionization efficiency of the target analytes. Therefore, minimizing matrix effects is critical to ensuring the accuracy of quantitative results obtained by this method [34].

In this study, blank Russula lepida was used as the matrix blank. Following the procedure described in Section 5.6, the matrix extract was used to prepare matrix-matched calibration curves, while reagent standard curves were prepared using 60% methanol aqueous solution as the solvent. The concentration levels of the 10 mushroom toxins in both types of calibration curves were prepared in accordance with Section 5.3.1 and Section 5.6. After instrumental analysis, the slopes of the matrix-matched and solvent-based standard curves were compared, and matrix factors (MF) were calculated as the ratio of these slopes.

As shown in Table 3, the MF values for all 10 mushroom toxins ranged from 80.55% to 118.91%. According to the criteria described in Section 5.6, an MF within the range of 80–120% indicates negligible matrix effects. These results suggest that the use of graphitized multi-walled carbon nanotubes (G-MWCNTs) for sample purification effectively removes complex matrices from wild mushrooms, thereby minimizing matrix effects and improving the accuracy of quantitative measurements.

### 2.6. Method Recovery and Precision

According to the spiking protocol described in Section 5.5, recovery experiments were conducted at three concentration levels. Each level was tested in six replicates. After sample pretreatment, the spiked samples were analyzed using the established method, and the average recovery and relative standard deviation (RSD) for each concentration were calculated. Detailed results are presented in Table 4. For seven toxins—α-amanitin, β-amanitin, γ-amanitin, phalloidin, phallacidin, phallisacin, and muscarine—the recoveries at the low concentration level (0.05 mg/kg) ranged from 70.67% to 117.60%, with RSDs between 4.84% and 18.11%. At the medium concentration level (0.15 mg/kg), recoveries ranged from 82.57% to 109.44%, with RSDs between 5.57% and 9.91%. At the high concentration level (0.5 mg/kg), recoveries were between 87.29% and 115.37%, with RSDs from 2.55% to 8.45%.

For the other three toxins—orellanine, gyromitrin, and muscimol—the low-level spiking concentration was set at 0.5 mg/kg, yielding recoveries of 72.86% to 93.21% and RSDs between 7.10% and 9.01%. At the medium concentration (1 mg/kg), recoveries ranged from 82.11% to 108.14%, with RSDs of 3.83% to 5.82%. At the high concentration level (2 mg/kg), recoveries were between 90.79% and 97.94%, with RSDs of 2.13% to 6.70%. All ten mushroom toxins showed RSDs below 19%, and the recoveries at low, medium, and high spiking levels were all above 70%. These results comply with the performance criteria set by the European Commission Directorate-General for Health and Food Safety guidelines for analytical quality control and method validation in residue analysis of food and feed (SANTE/11312/2021) [35], which stipulate acceptable recoveries in the range of 60–120% and RSDs below 20%. Overall, the optimized method demonstrated good reproducibility and accuracy for the quantification of ten mushroom toxins in wild mushroom matrices, meeting the requirements for quantitative analysis.

### 2.7. Analysis of Real Samples

To evaluate the feasibility and applicability of the analytical method, this study selected areas with a high incidence of mushroom poisoning in Zunyi City, Guizhou Province (Tongzi County, Meitan County, and Renhuai City) as sampling sites. A total of 45 wild mushroom samples were collected, including suspected *Amanita phalloides*, *Amanita europaea*, suspected *Amanita virosa*, *Amanita pseudoporphyria*, *Amanita subjunquillea*, *Russula* spp., and *Boletus* spp. The detailed results are presented in Table 5. Among the samples morphologically identified as *Amanita phalloides*, α-amanitin (281.47 mg/kg), β-amanitin (50.07 mg/kg), γ-amanitin (3.11 mg/kg), phalloidin (445.10 mg/kg), and phallacidin (2.74 mg/kg) were detected, indicating that primary-level personnel have significantly improved their ability to identify amanita phalloides. phalloides based on morphology through public health training. Interestingly, amanitins were also detected in samples that did not morphologically resemble *Amanita*, including α-amanitin (113.68 mg/kg), β-amanitin (250.29 mg/kg), γ-amanitin (55.49 mg/kg), and phalloidin (61.18 mg/kg), suggesting a risk of misidentification when relying solely on morphological characteristics. Samples misidentified by the public as edible mushrooms were found to contain low doses of phalloidin (0.42 mg/kg) and phallacidin (0.032 mg/kg). Although the concentrations were lower than those found in amanita phalloides. phalloides, they still pose potential health risks. Additionally, muscarine was detected in mushroom samples with appearances similar to edible species. Specifically, samples resembling Macrolepiota procera contained 0.035 mg/kg, and those resembling *Termitomyces* spp. contained 0.82 mg/kg, indicating that certain edible-looking mushrooms may easily be confused with toxic species, leading to food poisoning. As shown in Table 5, the overall toxin-positive rate among the samples was 22.22%. Among the ten types of toxins tested, only gyromitrin was not detected, possibly due to the limited distribution of gyromitra esculenta in the sampling area and the absence of corresponding samples.

## 3. Discussion

Early and accurate diagnosis of mushroom poisoning is essential for effective treatment, as mushroom toxins are the key causative agents of intoxication. Determining the type of toxin ingested provides critical guidance for clinicians to establish tailored therapeutic strategies. However, current analytical methods face major obstacles: no single detection technique can comprehensively identify all mushroom toxins; sample matrices contain complex interfering substances; and conventional morphological identification carries a risk of misclassification, particularly in rural areas.

In this study, we developed a rapid detection system incorporating nanomaterial-based purification. The graphitized sp^2^ carbon layer of the material offers a large surface area and abundant π-electron clouds, enabling strong affinity toward analytes with conjugated structures or aromatic rings (such as amatoxins and phallotoxins) via π–π stacking, hydrophobic interactions, and van der Waals forces. Using optimized graphitized multi-walled carbon nanotubes (G-MWCNTs) as a pretreatment material, macromolecular interferents—including pigments, proteins, and polysaccharides—were effectively removed. The system achieved high sensitivity (LOD 5–200 μg/kg), reduced analysis time to less than 60 min per sample, and maintained good tolerance to matrix effects (recovery 70.67–117.60%, RSD < 18.11%). These results indicate that the method can meet the requirements of resource-limited regions for mushroom toxin detection, although challenges such as solvent compatibility across diverse compounds and difficulties in pretreating complex samples remain unresolved.

The choice of extraction solvents in mushroom toxin analysis is guided by the chemical characteristics of the toxins, such as polarity, hydrophobicity, and their interactions with the sample matrix. Because mushroom toxins vary greatly in their chemical structures and physicochemical properties, and because sample matrices are highly heterogeneous, solvent selection must be tailored to the solubility of the target compound and the matrix composition. As a result, no universal extraction solvent system that covers all mushroom toxins and sample types has yet been established.

In this study, based on the diversity of mushroom toxins—including their polarity, nonpolarity, and molecular weight range (114–919 Da)—we used a 60% methanol aqueous solution as the extraction reagent. Although this solution, together with other commonly employed organic solvents such as acetonitrile and dichloromethane, provides high extraction efficiency, their volatility poses potential long-term health risks to laboratory staff. Alternatively, some studies have employed safer, more environmentally friendly solvents, such as water or PBS, which improve operational safety [36]. However, these methods are limited in toxin coverage (typically only 2–3 toxins) and show poor extraction efficiency for nonpolar toxins, thereby reducing detection sensitivity. Considering the diversity and physicochemical variability of mushroom toxins (e.g., psilocybin is water-soluble, whereas 2-amino-4,5-hexadienoic acid is thermally unstable), future research should focus on developing broad-spectrum, efficient, and environmentally sustainable extraction systems—such as deep eutectic solvents or modified buffer solutions—that balance sensitivity and laboratory safety.

In mushroom toxin analysis, purification of the extract to remove complex matrix interferences is a key step for ensuring reliable results. At present, solid-phase extraction (SPE) and dispersive solid-phase extraction (dSPE) are the most widely used purification techniques. In this study, we applied a dSPE approach using graphitized multi-walled carbon nanotubes (G-MWCNTs) as the purification material. This strategy provided effective cleanup while simplifying the workflow. As summarized in Table 6, the method enabled simultaneous analysis of ten mushroom toxins from four major classes, with detection limits ranging from 5 to 200 μg/kg, which are comparable to values reported in previous studies [37,38].

Each purification method has its strengths. SPE offers high selectivity and reproducibility, allowing efficient purification and enrichment of analytes, but the process is relatively time-consuming [17]. In contrast, dSPE requires fewer steps and is easier to operate. Recent studies have reported that optimized SPE protocols using PRiME HLB cartridges or Oasis PRiME HLB 96-well µElution Plates improved sample preparation efficiency and achieved high sensitivity and recovery for selected toxins [39]. However, the relatively high cost of these commercial products limits their use in resource-limited settings.

Although UHPLC–MS/MS assays targeting muscarine may achieve lower LODs for specific toxins, our method offers greater throughput, broader analyte coverage, and practicality. These features make it particularly suitable for real-world monitoring of wild mushrooms, where the toxin profile and sample matrices are often unknown and diverse. This provides a robust technical tool for public health monitoring and emergency response. Future studies should investigate the combined use of different sorbent materials to further improve efficiency, cost-effectiveness, and ease of operation, with the ultimate goal of establishing pretreatment workflows that are sensitive, economical, and practical.

The G-MWCNTs-based dSPE pretreatment method developed in this study demonstrated effective matrix cleanup for wild mushroom samples. However, several practical challenges deserve attention. First, samples may originate from mushrooms that have already been cooked. In preliminary experiments, we compared boiled and fresh mushrooms. Even after boiling for 1 h and refrigerated storage for one day, α-amatoxin (174.20 μg/kg) and phalloidin (334.72 μg/kg) were still detectable, indicating that boiling has minimal impact on the stability of these key toxins. In contrast, other cooking methods such as frying or stir-frying may cause more complex changes to the sample matrix and toxin stability. High-temperature oil processing, for instance, can alter lipids and proteins, producing new interfering compounds (e.g., Maillard reaction products) and aggravating matrix effects.

Second, method optimization showed that the dosage of G-MWCNTs has a dual influence on purification. While moderate amounts (50–150 mg) enhanced matrix cleanup, excessive use (>200 mg) led to over-adsorption of phallotoxins, reducing recoveries to 43.1–76.8%. These findings highlight the need to establish standardized pretreatment strategies that account for different cooking methods and to further investigate how complex matrices interfere with mass spectrometry detection. For heavily processed samples, applying isotope-labeled internal standards to correct recoveries, combined with secondary purification using SPE cartridges, may further improve accuracy. Overall, this study provides a practical framework for mushroom toxin detection. Nevertheless, validation with larger sample sets is still needed to ensure reliability when applied to complex cooked samples.

In this study, Orbitrap high-resolution mass spectrometry (HRMS) significantly improved qualitative confirmation for both toxin identification and screening. The mass deviations of all target analytes were below 0.75 ppm (Table 1), well within the conventional acceptance criterion (<5 ppm), thereby minimizing false positives from isotopic or isobaric interferences in complex matrices [20]. Furthermore, the data-dependent acquisition (dd-MS^2^) mode provided high-resolution fragment ion spectra in addition to accurate precursor masses, offering dual confirmation and increasing confidence in compound identification (Table 1). These results are consistent with the findings of Barbosa et al., who emphasized that LC-HRMS is the most reliable tool for amatoxin detection in complex matrices [17].

Although the quantification limits of this method (0.015–0.6 mg/kg) are comparable to, or slightly higher than, those obtained with triple quadrupole–based approaches [23,24,39], its strength lies in non-targeted screening. Full-scan data acquisition enables retrospective analysis, offering opportunities to detect unexpected or previously unknown mushroom toxin derivatives.

The 10-toxin simultaneous detection platform developed in this study provides critical technical support for strengthening mushroom toxin screening networks. Its cost-effectiveness and rapid turnaround make it suitable for implementation in wild mushroom–rich regions of southwest China. Unlike morphology-based identification requiring senior experts or DNA sequencing approaches, our method keeps the cost per sample below USD 6 and involves a straightforward pretreatment procedure. These advantages make it particularly suitable for laboratories with limited resources, improving emergency response capacity and offering real-time risk alerts for local residents consuming wild mushrooms. In addition, the 10-toxin detection system established here lays the groundwork for broader toxin monitoring.

Looking ahead, future research should expand toxin coverage to include neurotoxins (e.g., psilocybin) and nephrotoxic agents (e.g., 2-amino-4,5-hexadienoic acid). Further, the development of non-targeted screening methods using Orbitrap HRMS full-scan data combined with intelligent matching via the mzCloud database could enable the identification of unknown toxins. Finally, building a “Chinese Mushroom Toxin HRMS Database” that integrates accurate mass data (error < 3 ppm), retention times, characteristic fragments, and ion ratios would provide a robust foundation for non-targeted screening and the discovery of novel toxins.

## 4. Conclusions

In this study, a method was established for the simultaneous determination of ten mushroom toxins in wild mushroom species using ultra-performance liquid chromatography coupled with quadrupole/electrostatic field orbitrap high-resolution mass spectrometry (UPLC-Q Orbitrap HRMS), with graphitized multi-walled carbon nanotubes (G-MWCNTs) employed as the purification sorbent. The selection of G-MWCNTs was based on their unique multiple adsorption properties, including π–π conjugation, hydrophobic interactions, and large specific surface area. These features enable the efficient removal of organic interferences such as pigments and lipids, as well as selective elimination of biomacromolecules like proteins and polysaccharides [34]. Coupled with the UPLC-Q Orbitrap HRMS technique, the optimized method yielded satisfactory recovery rates and precision. The recovery ranged from 70.67% to 117.63%, with relative standard deviations (RSDs) between 2.13% and 18.11%. The method also demonstrated good sensitivity, with limits of quantification (LOQs) ranging from 0.015 to 0.6 mg/kg. Furthermore, the optimized method was applied to analyze 45 wild mushroom samples. Amanitin toxins were detected in the suspected lethal *Amanita* species, orellanine was identified in the suspected *Amanita subglobosa*, and muscarine was found in samples of presumed edible species such as *Termitomyces* and *Macrolepiota procera*. These findings confirm the applicability of the proposed method for detecting ten types of mushroom toxins in wild mushroom samples. Future studies will expand the detection scope to include additional toxins, such as 2-amino-4,5-hexadienoic acid potentially present in suspected *Amanita europaea* and *Amanita pseudoporphyria* species. Therefore, the G-MWCNTs-based UPLC-Q Orbitrap HRMS method proposed in this study is simple, convenient, and efficient, making it suitable for the determination of ten common mushroom toxins in wild mushrooms. The application of this method provides a reliable technical support for accurate detection of multiple mushroom toxins in complex biological matrices, and offers a dependable analytical tool for food safety risk monitoring and public health incident response.

## 5. Materials and Methods

### 5.1. Instruments and Equipment

An Orbitrap 120 ultra-high-performance liquid chromatography–quadrupole–electrostatic field Orbitrap high-resolution mass spectrometer (Thermo Fisher Scientific, Waltham, MA, USA) was used for analysis. A Multi Reax vortex shaker (Heidolph, Schwabach, Germany) and a Milli-Q ultrapure water system (Millipore, Burlington, MA, USA) were used during sample preparation. A 54-position XcelVAP nitrogen evaporator (Horizon Technology, Lake Forest, CA, USA), a TGL-16A high-speed refrigerated centrifuge (Pingfan Technology Co., Ltd., Changsha, China), and a Mettler Toledo XSR205 electronic balance (Mettler-Toledo, Parramatta, Switzerland) were employed for sample processing. A ZXRD-7080 blast drying oven (Zhicheng Analytical Instrument Manufacturing Co., Ltd., Shanghai, China), an HM100 knife mill (Beijing Grinder Instrument Co., Ltd., Beijing, China), and an SYU-30-900-DT ultrasonic cleaner (Shengyuan Instrument Co., Ltd., Zhengzhou, China) were also used. Organic phase syringe filters with 0.22 μm pore size (Anpu Experimental Technology Co., Ltd., Shanghai, China) were applied for sample filtration.

### 5.2. Materials and Reagents

Methanol and acetonitrile (both LC-MS grade) were purchased from Thermo Fisher Scientific (USA). Formic acid (LC-MS grade) was obtained from Anpu Experimental Technology Co., Ltd. (Shanghai, China), and ammonia solution (analytical grade) was obtained from Tianjin Comeo Chemical Reagent Co., Ltd. (Tianjin, China). Graphitized multi-walled carbon nanotubes (G-MWCNTs) with a particle diameter of 20–30 nm and a length of 5–30 μm were purchased from Aladdin Biochemical Technology Co., Ltd. (Shanghai, China). Ultrapure water (resistivity: 18.2 MΩ·cm) was produced using a Milli-Q purification system.

Standard substances for the 10 mushroom toxins were as follows: α-Amanitin (CAS: 23109-05-9), β-Amanitin (CAS: 21150-22-1), γ-Amanitin (CAS: 21150-23-2), phalloidin (CAS: 17466-45-4), phallacidin (CAS: 26645-35-2), and phallisacin (CAS: 58286-46-7) were purchased from Qingdao Pribolab Biological Engineering Co., Ltd. (Qingdao, China). orellanine (CAS: 37338-80-0) and gyromitrin (CAS: 16568-02-8) were obtained from Tianjin Alta Technology Co., Ltd. (Tianjin, China). Muscarine iodide (CAS: 24570-49-8) and muscimol (CAS: 2763-96-4) were purchased from Anpu Technology Co., Ltd. (Shanghai, China).

### 5.3. Solution Preparation

#### 5.3.1. Preparation of Standard Solutions

Muscarine iodide (2 mg, solid) and muscimol (1 mg, solid) were each accurately dissolved in 1.0 mL of methanol to prepare stock solutions with concentrations of 2 mg/mL and 1 mg/mL, respectively. These solutions were stored in the dark at temperatures below −18 °C. Commercially obtained standard solutions of α-amanitin, β-amanitin, γ-amanitin, phalloidin, phallacidin, phallisacin, and orellanine had a concentration of 50 mg/L, while the concentration of the gyromitrin standard solution was 100 mg/L. According to experimental requirements, a mixed standard working solution of the 10 mushroom toxins was prepared using methanol as the diluent. The concentrations were as follows: 2 mg/L for α-amanitin, β-amanitin, γ-amanitin, phalloidin, phallacidin, phallisacin, and muscarine; 10 mg/L for orellanine, gyromitrin, and muscimol.

Mixed standard working solutions at varying concentrations were prepared by diluting the mixed standard solution with 60% methanol–water (*v*/*v*). The final concentrations were as follows: For α-amanitin, β-amanitin, γ-amanitin, phalloidin, phallacidin, phallisacin, and muscarine: 0.005 mg/L, 0.01 mg/L, 0.02 mg/L, 0.05 mg/L, 0.1 mg/L, 0.2 mg/L, 0.5 mg/L, and 1 mg/L. For orellanine, gyromitrin, and muscimol:0.05 mg/L, 0.1 mg/L, 0.2 mg/L, 0.5 mg/L, 1 mg/L, 2 mg/L, 3 mg/L, and 5 mg/L.

#### 5.3.2. Methanol Aqueous Solution, 60%

Accurately pipette 60 mL of methanol and dilute to 100 mL with ultrapure water.

#### 5.3.3. Mixed Aqueous Solution of 0.1% Formic Acid and 0.01% Ammonia

Add 1 mL of formic acid and 0.1 mL of ammonia to 1000 mL of ultrapure water, then mix well.

### 5.4. Sample Pretreatment

Fresh wild mushroom samples were dried in a thermostatic drying oven at 45 °C for 18 h. The dried samples were then ground using a pulverizer, thoroughly homogenized, and stored in clean, sealed containers. An accurately weighed 1.0 g portion of the ground sample was transferred into a 15 mL centrifuge tube, followed by the addition of 10 mL of 60% methanol aqueous solution. After vortexing for 3 min, the mixture was subjected to ultrasonic extraction for 10 min and then centrifuged at 10,000 r/min for 5 min. The entire supernatant was transferred to another 15 mL centrifuge tube containing 150 mg of G-MWCNTs sorbent, vortexed for 3 min, and centrifuged again at 10,000 r/min for 5 min. The resulting supernatant was transferred to a new 15 mL centrifuge tube and evaporated to near dryness in a 40 °C water bath under a nitrogen stream. The residue was reconstituted with 1.0 mL of 60% methanol aqueous solution, vortexed for 1 min, and filtered through a 0.22 μm syringe filter. The final extract was analyzed using ultra-high-performance liquid chromatography coupled with quadrupole/orbitrap high-resolution mass spectrometry (UHPLC-Q-Orbitrap HRMS).

### 5.5. Spiking Experiments

Under the optimized chromatographic and mass spectrometric conditions, Russula lepida was used as a blank sample. A mixed standard solution containing seven toxins—α-amanitin, β-amanitin, γ-amanitin, phallacidin, phallisacin, phalloidin, and muscarine—was spiked into the blank samples at three concentration levels: 50, 150, and 500 μg/kg. In parallel, a mixed standard solution of three additional toxins—orellanine, gyromitrin, and muscimol—was spiked into the blank samples at three concentration levels: 0.5, 1, and 2 mg/kg. Each concentration level was tested in six replicates to calculate the spiked recovery rates and method precision. To determine the method detection limit (LOD) and quantification limit (LOQ), an appropriate amount of mixed standard solution was added to the blank Russula lepida samples, followed by sample pretreatment and analysis. The LOD and LOQ were defined as the concentrations corresponding to signal-to-noise ratios (S/N) of 3 and 10, respectively [40].

### 5.6. Matrix Effect

The matrix effect (ME) was evaluated by calculating the matrix factor (MF), which is defined as the ratio of the slope of the matrix-matched calibration curve—prepared using blank extracts after purification—to the slope of the calibration curve prepared in pure solvent. In general, an MF value close to 100% (i.e., within the range of 80–120%) indicates no or negligible matrix effect. MF values between 50–80% or 120–150% are considered to represent moderate matrix effects, while values greater than 150% or less than 50% are indicative of strong matrix effects [41]. Russula lepida was used as the blank matrix. After purification and extraction following the procedure described in Section 5.4, matrix-matched calibration standards were prepared at concentrations ranging from 0.005 to 5 mg/L using the blank extract as the solvent. Similarly, a standard calibration curve was prepared using 60% methanol as the solvent at the same concentration range. Both sets of standards were analyzed under the same instrumental conditions.

### 5.7. Instrumentation Conditions

#### 5.7.1. Chromatographic Conditions

Chromatographic separation was performed on a Hypersil GOLD C18 column (100 mm × 2.1 mm, 1.9 μm particle size). The mobile phase flow rate was set at 0.2 mL/min. The column temperature was maintained at 35 °C, and the autosampler temperature was set to 10 °C. The injection volume was 2 μL. The mobile phase A consisted of 0.1% formic acid and 0.01% ammonium hydroxide in water, while mobile phase B was acetonitrile. A linear gradient elution was applied as follows: 0–0.5 min, 5% B; 0.5–5.5 min, 60% B; 5.5–7.0 min, 95% B; 7.1–9.0 min, 5% B.

#### 5.7.2. Mass Spectrometry Conditions

An Orbitrap 120 high-resolution mass spectrometer equipped with a heated electrospray ionization (H-ESI) source was used. Data were acquired in polarity-switching mode (positive and negative ion modes). The ion transfer tube temperature was set at 300 °C. The spray voltage was 3500 V for positive mode and 2800 V for negative mode. Sheath gas (N_2_) pressure was 40 Arb, auxiliary gas pressure was 8 Arb, and sweep gas pressure was 1 Arb. The vaporizer temperature was set to 400 °C. Full MS scans were acquired over the *m*/*z* range of 62–930 with a resolution of 60,000. The automatic gain control (AGC) target was set to standard mode. The scan mode was Full MS/dd-MS^2^. Internal mass calibration was performed using the EASY-IC source. The resolution for MS^2^ was set to 30,000, with collision energies of 20%, 40%, 50%, 60%, and 90%. Apex trigger was enabled at 30%.

## Figures and Tables

**Table 1:** Retention time and mass spectrum information of 10 mushroom toxins.

Compound	Molecular Formula	Retention Time	Precursor Ion (*m*/*z*)			Production (*m*/*z*)	
			Theoretical Value	Measured Value	Mass Error (10^−6^)	Fragment 1	Fragment 2
α-Amatoxin	C_39_H_54_N_10_O_14_S	4.11	917.34689	917.34753	0.70222	899.33533	201.07072
β-Amatoxin	C_39_H_53_N_9_O_15_S	4.09	918.33091	918.33081	0.10830	900.31924	205.04417
γ-Amatoxin	C_39_H_54_N_10_O_13_S	4.42	901.35198	901.35211	0.14624	883.34033	205.04390
Phalloidin	C_35_H_48_N_8_O_11_S	4.79	789.3236	789.32336	0.29866	86.06004	157.07602
Phallacidin	C_37_H_50_N_8_O_13_S	4.90	847.32908	847.32892	0.19065	811.30795	157.07602
Phallisacin	C_37_H_50_N_8_O_14_S	4.61	863.32400	863.32465	0.74827	130.06513	157.07602
Orellanine	C_10_H_8_N_2_O_6_	3.62	253.04551	253.04546	0.21367	191.04530	219.04015
Gyromitrin	C_4_H_8_N_2_O	3.53	101.07094	101.07087	0.69807	58.02874	42.03383
Muscarine	C_9_H_20_NO_2_	1.74	174.14886	174.14882	0.23560	97.06479	57.03349
Muscimol	C_4_H_6_N_2_O_2_	1.41	115.0502	115.05016	0.38557	98.02365	67.01784

**Table 2:** Linear equations, linear ranges, R^2^, LOD and LOQ of 10 mushroom toxins.

Compound	Linear Range (mg·L^−1^)	Regression Equation	R^2^	LOD (mg·kg^−1^)	LOQ (mg·kg^−1^)
α-Amatoxin	0.02–1	y = 2.711 × 10^4^x − 2.384 × 10^2^	0.9985	0.02	0.06
β-Amatoxin	0.01–1	y = 1.246 × 10^5^x − 6.919 × 10^3^	0.9969	0.015	0.045
γ-Amatoxin	0.02–1	y = 6.410 × 10^4^x − 2.007 × 10^4^	0.9954	0.02	0.06
Phalloidin	0.01–1	y = 7.517 × 10^4^x − 8.747 × 10^3^	0.9937	0.015	0.045
Phallacidin	0.02–1	y = 2.876 × 10^4^x + 6.787 × 10^3^	0.9989	0.02	0.06
Phallisacin	0.02–1	y = 2.778 × 10^4^x + 6.403 × 10^3^	0.9986	0.02	0.06
Orellanine	0.2–5	y = 5.197 × 10^3^ x + 2.597 × 10^3^	0.9940	0.2	0.6
Gyromitrin	0.05–5	y = 3.199 × 10^4^ x − 2.026 × 10^3^	0.9936	0.1	0.3
Muscarine	0.005–1	y = 2.655 × 10^5^x − 9.613 × 10^3^	0.9952	0.005	0.015
Muscimol	0.2–5	y = 3.891 × 10^3^x + 2.709 × 10^3^	0.9977	0.2	0.6

**Table 3:** Matrix effects of 10 mushroom toxins.

Compound	Pure Solvent	R^2^	Sample Extract with Treatment	R^2^	MF (%)
	Y = K_1_X + b_1_		Y = K_2_X + b_2_		
α-Amatoxin	Y = 1.021 × 10^4^X − 7.615 × 10^4^	0.9996	Y = 9.307 × 10^3^X + 3.454 × 10^3^	0.9977	91.16
β-Amatoxin	Y = 4.579 × 10^4^X − 4.430 × 10^3^	0.9992	Y = 5.076 × 10^4^X − 4.429 × 10^3^	0.9928	110.85
γ-Amatoxin	Y = 2.605 × 10^4^X − 3.062 × 10^3^	0.9987	Y = 2.337 × 10^4^X + 3.673 × 10^3^	0.9993	89.71
Phalloidin	Y = 6.766 × 10^4^X − 7.205 × 10^3^	0.9993	Y = 7.073 × 10^4^X + 1.012 × 10^3^	0.9992	104.54
Phallacidin	Y = 1.419 × 10^4^X − 3.109 × 10^3^	0.9907	Y = 1.143 × 10^4^X − 5.932 × 10^4^	0.9924	80.55
Phallisacin	Y = 3.586 × 10^4^X + 8.618 × 10^3^	0.9965	Y = 3.812 × 10^4^X − 6.792 × 10^3^	0.9971	106.30
Orellanine	Y = 5.953 × 10^3^X + 8.633 × 10^3^	0.9945	Y = 5.770 × 10^3^X + 3.611 × 10^3^	0.9970	96.93
Gyromitrin	Y = 4.084 × 10^4^X − 1.963 × 10^4^	0.9992	Y = 4.519 × 10^4^X + 5.8261 × 10^3^	0.9983	110.65
Muscarine	Y = 4.092 × 10^5^X − 4.213 × 10^4^	0.9919	Y = 3.794 × 10^5^X + 8.356 × 10^3^	0.9990	92.72
Muscimol	Y = 4.590 × 10^3^X − 1.483 × 10^3^	0.9902	Y = 5.458 × 10^3^X + 9.195 × 10^3^	0.9932	118.91

**Table 4:** The average recovery and precision of 10 mushroom toxins (*n* = 6).

Compound	Added/(mg·kg^−1^)	Recovery/%	RSD/% (*n* = 6)
α-Amatoxin	0.05, 0.15, 0.5	70.67, 82.57, 87.29	18.11, 9.91, 8.45
β-Amatoxin	0.05, 0.15, 0.5	117.60, 104.72, 106.31	11.12, 5.75, 7.13
γ-Amatoxin	0.05, 0.15, 0.5	106.87, 103.65, 93.46	8.72, 5.76, 6.04
Phalloidin	0.05, 0.15, 0.5	75.16, 86.47, 97.76	10.81, 7.33, 7.18
Phallacidin	0.05, 0.15, 0.5	76.82, 91.68, 101.83	12.01, 7.48, 5.23
Phallisacin	0.05, 0.15, 0.5	90.68, 95.81, 102.85	6.83, 7.25, 5.14
Orellanine	0.5, 1, 2	72.86, 82.11, 90.79	8.15, 5.82, 2.13
Gyromitrin	0.5, 1, 2	93.21, 104.84, 96.05	7.10, 3.83, 6.70
Muscarine	0.05, 0.15, 0.5	91.89, 109.44, 115.37	4.84, 5.57, 2.55
Muscimol	0.5, 1, 2	87.35, 108.14, 97.94	9.01, 4.90, 4.36

**Table 5:** Detection results of 10 mushroom toxins in wild mushrooms.

Sample Serial Number	Detection Results (mg/kg)									
	α-Amatoxin	β-Amatoxin	γ-Amatoxin	Phalloidin	Phallacidin	Phallisacin	Orellanine	Gyromitrin	Muscarine	Muscimol
1	113.68	250.29	55.49	61.18	ND	ND	ND	ND	ND	ND
2	281.47	50.07	3.11	445.10	2.74	ND	ND	ND	ND	ND
3	ND	ND	ND	0.42	0.032	ND	ND	ND	ND	ND
4	ND	ND	ND	ND	ND	ND	1.24	ND	ND	ND
5	ND	ND	ND	ND	ND	ND	ND	ND	0.035	ND
6	ND	ND	ND	ND	ND	ND	ND	ND	0.82	ND
7	ND	ND	ND	ND	ND	ND	ND	ND	0.54	ND
8	ND	ND	ND	ND	ND	ND	ND	ND	0.57	ND
9	ND	ND	ND	ND	ND	ND	ND	ND	ND	55.05
10	ND	ND	ND	1.77	ND	ND	ND	ND	ND	119.55

Note: ND indicates not detected.

**Table 6:** Comparison of various methods for the determination of mushroom toxins.

Method	Retention Time (min)	Number of Detected Toxins	LOD/(µg kg^−1^)	Purification Material	Matrix Effects (%)	Sample Purification Cost (USD)	Processing Time	References
HPLC-UV-EC	18.1	2	24–64	HLB-SPE	NA	3	3 h	[2]
UHPLC-Q-Orbitrap MS	13.5	4	8–20	NA	48–101	NA	1 h	[20]
LC-QqQ_LIT_-MS/MS	15	2	100–1000	QuCHERS-PP column	NA	2.8	2 h	[37]
LC-TOF-MS/MS	22.3	9	9.8–4900	HLB-SPE	NA	3	2 h	[38]
UHPLC-MS/MS	8	12	5–100	NA	38–66	NA	1 h	[39]
UHPLC-Q Orbitrap HRMS	9	10	5–200	G-MWCNTs	80–119	1.8	1 h	This work

Note: “NA” indicates not provided.

## Data Availability

The original contributions presented in this study are included in the article. Further inquiries can be directed to the corresponding author.
